# Controlled preparation of a MCC-*g*-AM/EDA/PA loaded Fe(iii) adsorbent by the pre-radiation grafting method and its application for the adsorption removal of phosphate

**DOI:** 10.1039/d0ra09389k

**Published:** 2021-02-03

**Authors:** Yuan Zhao, Jin-Yu Yang, Ting-Ting Li, Guo Liu, Ya-Yang Wang, Yu Jiang, Zheng-Xi Wang, Fang-Fang Zhang, Yue-Sheng Li, Yi Liu

**Affiliations:** Key Laboratory of Coal Conversion, New Carbon Materials of Hubei Province, School of Chemistry and Chemical Engineering, Wuhan University of Science and Technology Hubei Wuhan 430081 China frank78929@163.com yiliuchem@whu.edu.cn; Non-power Nuclear Technology Collaborative Innovation Center, Hubei Key Laboratory of Radiation Chemistry and Functional Materials, Hubei University of Science & Technology Hubei Xianning 437100 P. R. China; State Key Laboratory of Separation Membranes and Membrane Processes, College of Chemistry and Chemical Engineering, College of Environmental Science and Engineering, Tiangong University Tianjin 300387 P. R. China

## Abstract

A new spherical cellulose-based adsorbent and high phosphate removal rate microcrystalline cellulose-*g*-acrylamide/ethylenediamine/phthalic anhydride (MCC-*g*-AM/EDA/PA) loaded Fe(iii) adsorbent was prepared by a pre-radiation grafting and chemical modification method. Fe(iii) was successfully introduced into the modified grafted chains of the MCC-*g*-AM/EDA/PA resin, and characterized by FTIR, TG, XRD, SEM and XPS. The optimized conditions for the grafting reaction of acrylamide (AM) onto MCC were 20% AM emulsion at an absorbed dose of 30 kGy, and a grafting rate as high as 247%. In addition, the adsorption performance of the adsorbent was tested by static adsorption experiments with phosphate. The adsorbent resin showed excellent adsorption performance under alkaline conditions, contributions to the synergetic effect of precipitation, and inner-sphere surface complex reactions. The adsorption efficiency can reach 89.7% at low concentration. In summary, the neotype spherical cellulose-based adsorbent has the advantages of being separation-free in bulk materials, avoiding secondary pollution, and being easy to recycle, and it could be employed as an environmentally friendly adsorbent for phosphate removal in eutrophic water.

## Abstract 2

A new spherical cellulose-based adsorbent and high phosphate removal rate microcrystalline cellulose-*g*-acrylamide/ethylenediamine/phthalic anhydride (MCC-*g*-AM/EDA/PA) loaded Fe(iii) adsorbent was prepared by a pre-radiation grafting and chemical modification method.

## Graphical abstract

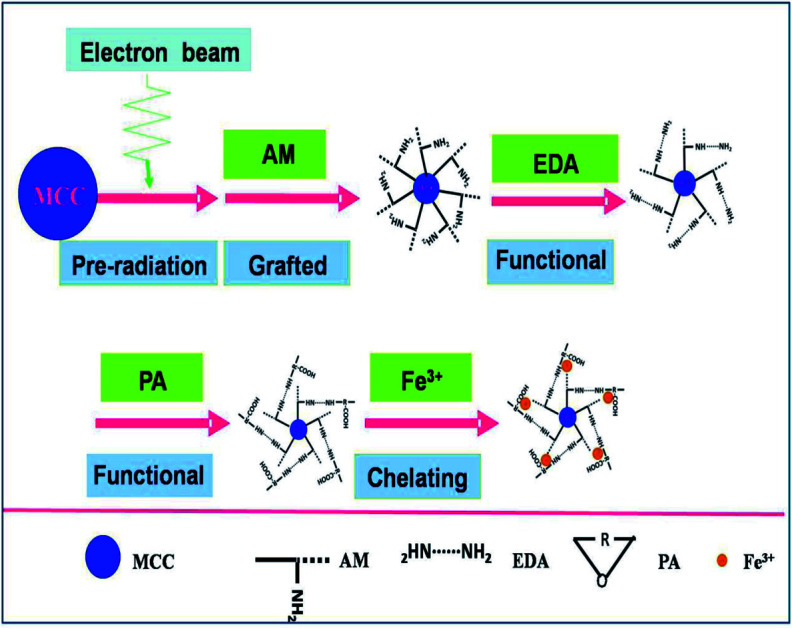

## Introduction

The rapid pace of industrialization and tremendous increase of the human population in the last few decades have caused serious environmental pollution. Phosphate, as one of the essential restrictive nutrient elements, can promote the growth of organisms in water bodies.^1,2^ It has a positive effect on plant growth, but excessive phosphate results in water eutrophication, so phosphate pollution has been extensively investigated by relevant researchers.^3,4^ Previous studies have shown that the phenomenon of rapid propagation of algae and other plankton, reduced dissolved oxygen, and large-scale fish and other biological deaths may be caused by phosphate eutrophication.^5^

In recent years, several treatment technologies have been applied for the removal of pollutants from aqueous solution, including adsorption, photo-catalysis, chemical oxidation and reduction, biological degradation, electrochemical treatment, and coagulation.^6,7^ Among these approaches, adsorption is considered as a promising technology for industrial applications due to its low-cost, low sludge production, and simple operation.^8^

Therefore, it is necessary to develop a new type of highly efficient, cleaner and less costly adsorbent to explore phosphate removal techniques, so as to solve the effective control of phosphate emissions leading to eutrophication problems.^9^

Microcrystalline cellulose (MCC) is argued to be the most abundant natural renewable material, and has been receiving great attention as a carrier matrix, due to its hydrophilicity, bio-compatibility, and abundance in nature.^10^ MCC possesses a highly crystalline structure consisting of repeating units of glucose linked via-1,4 glycosidic bonds, allowing easy generation of free radicals, and affording the ability to trigger other reactions.^11^ Some novel adsorbents based on MCC have been synthesized by common chemical methods, and these exhibited good adsorption performances.^12–15^

Recently, ionizing radiation has been developed as a simple and efficient method to prepare various spherical polymer adsorbents.^16^ Some new types of adsorbents for phosphate removal have been investigated recently, such as Cu(ii)-loaded HDPE-*g*-PAAm film,^17^ quaternized DMAEMA grafted non-woven fabric,^18^ Cu(ii) loaded GMA-*g*-DPA-PP/PE fabrics,^19^ Fe(iii)-IDA fabric,^20^ and GMA/AMP-PE non-woven fabric.^21^ Radiation induced processes have been demonstrated to have distinctive advantages compared with common chemical methods, such as high efficiency, no requirement for initiators, no tricky control of the experimental conditions, and no limitations in trunk polymer shape.^22^ For this reason, intermediate MCC-*g*-AM microspheres can be prepared by an electron beam pre-radiation grafting method, and utilized as an initial substrate for subsequent chemical modification. In order to promote the adsorption of phosphate on the resin, the chemically modified resin will be chelated with Fe(iii), because Fe(iii) is a Lewis acid that preferentially bonds with phosphate (a Lewis base).

In this work, we report a new spherical cellulose-based MCC-*g*-AM/EDA/PA with Fe(iii) chelation (denoted as: MAF) adsorbent prepared by a pre-radiation grafting and chemical modification method for the removal of phosphate in aqueous solutions. The microstructure and surface micromorphology of all the adsorbent resins were characterized by FTIR, TG, XRD, SEM and XPS. The adsorption performance of the MAF adsorbent was tested by static adsorption experiments towards phosphate. At the same time, a possible adsorption mechanism of MAF has been proposed. It is confirmed further that MAF resin is facile for phosphate liberation and recycling, which is beneficial for water purification in real-world situations.

## Results and discussion

### Synthesis and characterization of the MAF adsorbent

**Fig. 1:**
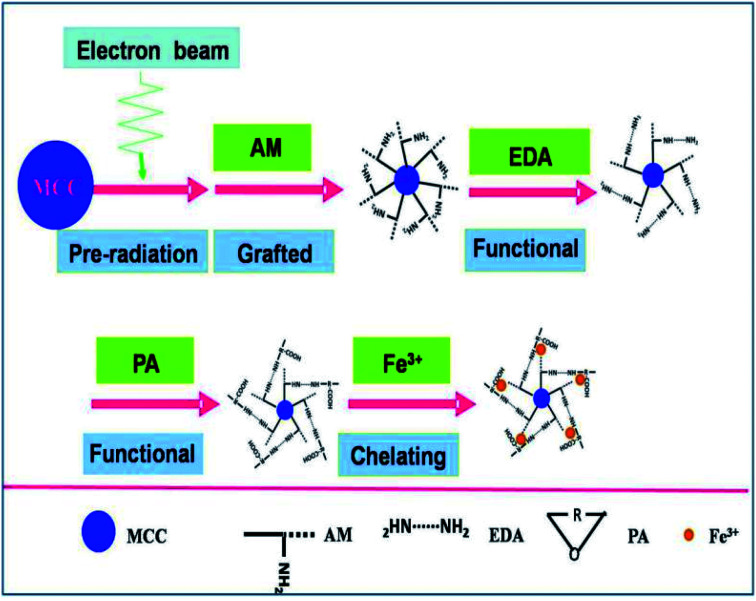
Scheme of the synthetic process of the MAF adsorbent resin.

The MAF adsorbent resin was prepared *via* electron beam radiation and chemical modification methods, as illustrated in Fig. 1. By exposure to electron beam radiation, the surface of the MCC decomposes, first to form a large number of trapped radicals, and then the acrylamide (AM) monomer was grafted onto the trapped radicals on the surface of the MCC under vacuum or in the presence of a nitrogen atmosphere. Subsequently, a specific secondary functional group was introduced into the MCC-*g*-AM resin through chemical treatment of the grafted functional group *via* refluxing with ethylenediamine (EDA) and phthalic anhydride (PA), as has been similarly reported in ref. ^20^. Finally, Fe(iii) was chelated into the adsorbent centers of MCC-*g*-AM/EDA/PA.

**Fig. 2:**
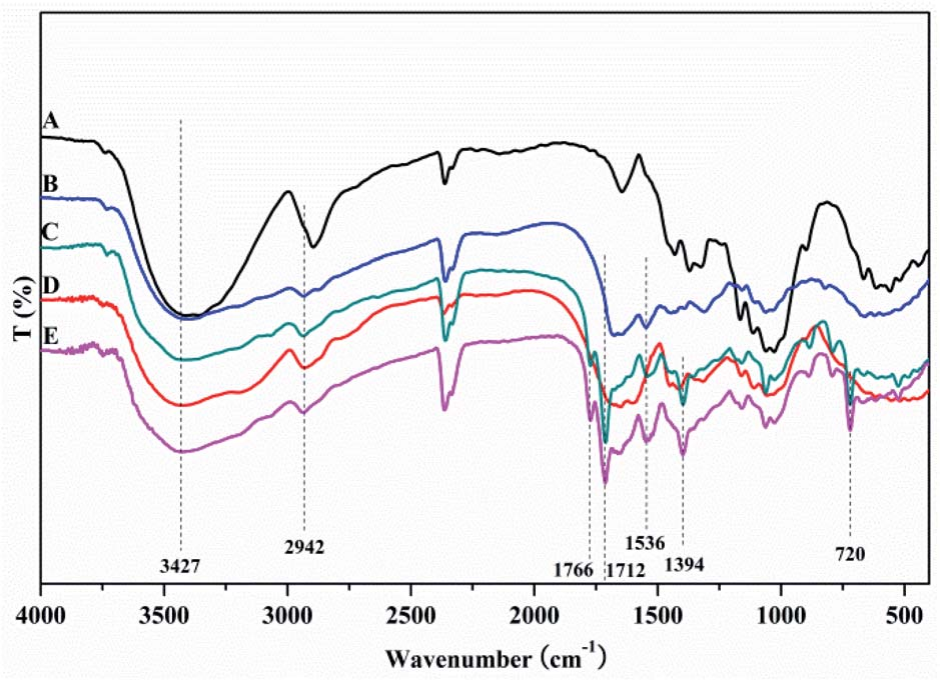
FTIR spectra of different adsorbents. A: MCC; B: MCC-*g*-AM; C: MCC-*g*-AM/EDA; D: MCC-*g*-AM/EDA/PA; E: MAF.

FTIR spectra of MCC, MCC-*g*-AM, MCC-*g*-AM/EDA, MCC-*g*-AM/EDA/PA and MAF are shown in Fig. 2. In the spectrum of MCC, the intense and broad band at 3200–3550 cm^−1^ is ascribed to the stretching vibrations of –OH, and that at 1450 cm^−1^ was assigned to CH–OH bending vibrations. After AM was grafted onto MCC, a new peak at 1595 cm^−1^ corresponding to N–H bending vibration (amine II band) was observed. Furthermore, the broad band at 1667 cm^−1^ was assigned to a carbonyl group (C=O, amine I band) of AM. Compared with the spectrum of MCC-*g*-AM, a new characteristic peak for MCC-*g*-AM/EDA appeared at 1536 cm^−1^, which was attributed to N–H groups of EDA. In the spectrum of MCC-*g*-AM/EDA/PA, the peaks at 1766 cm^−1^ and 1712 cm^−1^ were attributed to the C=O bond of the acid and the C=O bond of the amide, and the peaks at 1394 cm^−1^ and 720 cm^−1^ were attributed to the C=C and C–H stretching of the benzene ring. Thus, FTIR confirms the reaction progress of the preparation MCC-*g*-AM/EDA/PA. The characteristic peaks did not change significantly after Fe(iii) ion chelation into MCC-*g*-AM/EDA/PA.

**Fig. 3:**
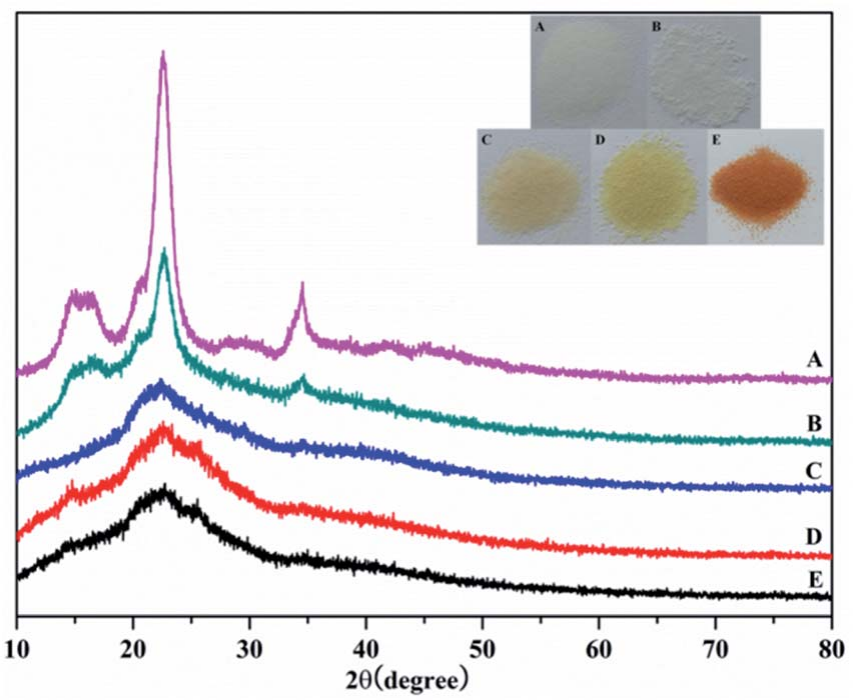
XRD patterns of different adsorbents. A: MCC; B: MCC-*g*-AM; C: MCC-*g*-AM/EDA; D: MCC-*g*-AM/EDA/PA; E: MAF; the inset is optical photos of the different adsorbents.

**Fig. 4:**
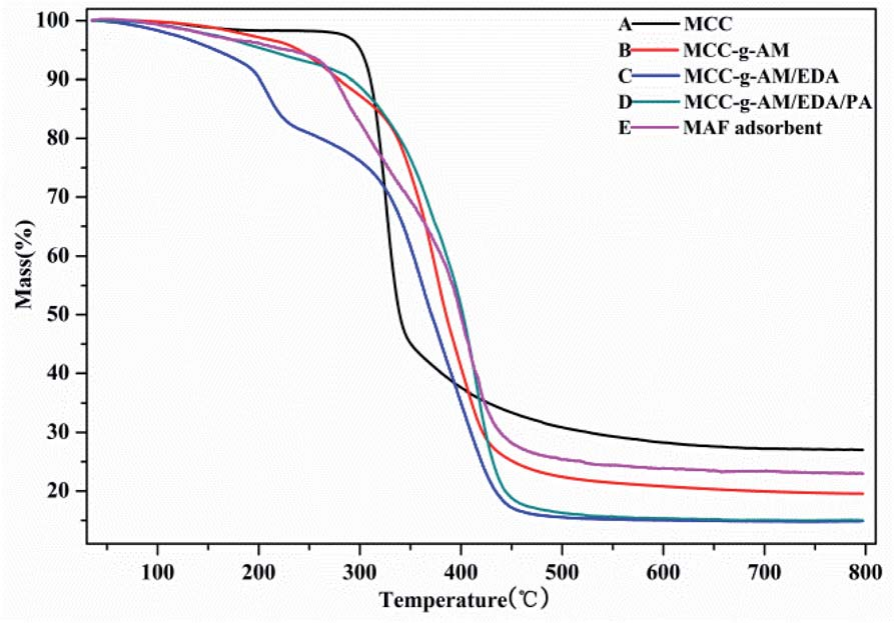
Thermo gravimetric analysis profiles of different adsorbents.

Fig. 3 shows the X-ray diffraction (XRD) analysis of the products. The diffraction peaks at 22.5° and 34.6° correspond to the 101 and 040 crystal planes of cellulose, respectively. Meanwhile, the weakening of these two diffraction peaks proved that graft polymerization had taken place in MCC. The weakening of the diffraction peak at 15° showed that graft polymerization increased the amorphous properties of the polymer. These results indicated that the cellulose had been grafted with AM, which was in agreement with the FTIR results described earlier. What's more, after chemical treatment with EDA and PA, the disappearance of the two diffraction peaks at 2*θ* of 15° and 34.6° was observed. The change in color supports each step of the reaction. As shown in the Fig. 4 inset, the initial MCC is white particles, but MCC-*g*-AM is milky white particles after AM was grafted onto the surface of MCC. In addition, the particle size is significantly increased. The color of MCC-*g*-AM/EDA and MCC-*g*-AM/EDA/PA gradually deepened after chemical treatment. Finally, the MAF adsorbent turned reddish brown, which may be due to the small amount of Fe(iii) ion oxidation to produce relatively stable Fe_2_O_3_ particles that adhere to the surface of the MAF adsorbent.

The thermal stability of these products was analyzed by TGA as shown in Fig. 4, the weight loss around 5% being slow and representing only water evaporation from room temperature to 200 °C. However, the weight loss was accelerated at 200–400 °C; this indicated that the temperature accelerated the decomposition of the grafted AM and chemical treatment of EDA and PA after 200 °C. Compared with the other samples, MCC showed a higher residual rate owing to the high carbon content. The residual rate of MAF appeared higher than that of MCC-*g*-AM/EDA/PA, which is most likely due to the Fe(iii) ion oxidation and the fact that Fe_2_O_3_ is relatively stable. The MAF adsorbent indicated that an inner-sphere surface complex (Fe–O–P) between phosphate and Fe_2_O_3_ metal oxides may be formed during the adsorption process, which may be the main reason for phosphate removal.^23^

**Fig. 5:**
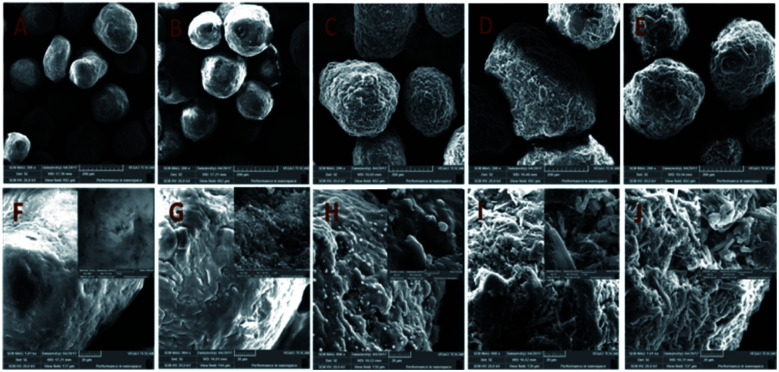
SEM images of different adsorbents. A and F: MCC; B and G: MCC-*g*-AM; C and H: MCC-*g*-AM/EDA; D and I: MCC-*g*-AM/EDA/PA; E and J: MAF.

The surface morphology and the properties of MCC, MCC-*g*-AM, MCC-*g*-AM/EDA, MCC-*g*-AM/EDA/PA and MAF were subjected to SEM analysis, as shown in Fig. 5A. The primary particle size of MCC is 200 ± 20 μm with uneven sizes. There are no visible holes or cracks, and the surface of the microspheres is very smooth, as was confirmed through the partial enlarged detail [Fig. 5F]. However, the microsphere diameter of MCC-*g*-AM/EDA/PA increased dramatically after AM, EDA and PA were introduced into the MCC. Moreover, the surface of the resin becomes rough owing to the chemical treatment and the existence of the gaps and holes results in a larger surfaces area, and this has great significance for its adsorption performance.^24^ Meanwhile, the morphology of MAF did not change significantly in comparison with MCC-*g*-AM/EDA/PA after chelating with Fe(iii) ions.

**Fig. 6:**
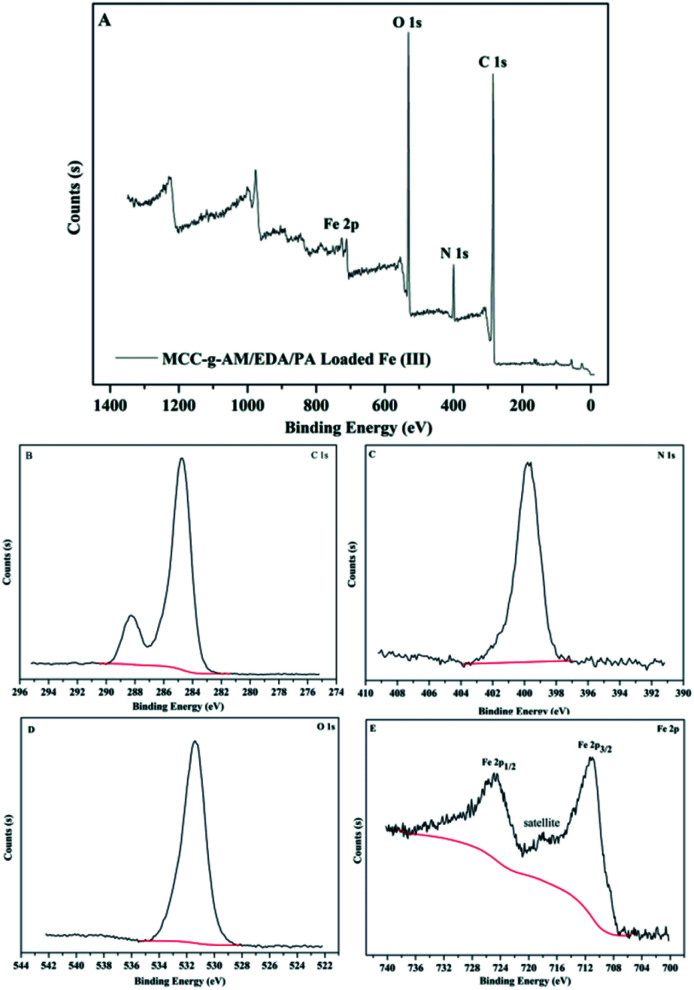
XPS spectra of MAF. (A) Global range X-ray photoelectron spectra; (B) C 1s; (C) N 1s; (D) O 1s; (E) Fe 2p.

**Fig. 7:**
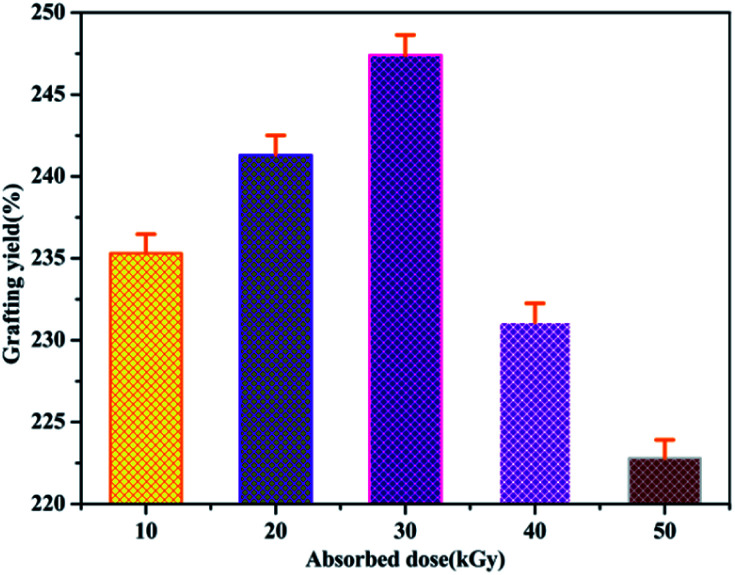
Grafting yield of MCC-*g*-AM under different absorbed doses.

It is well known that XPS analysis is a useful tool to verify the elemental composition of compounds. The XPS spectra of MAF are presented in Fig. 6. There were four primary peaks attributed to C1s, N1s, O1s and Fe2p at the BE of 284.74 eV, 399.75 eV, 531.41 eV and 711.23 eV, respectively. Fig. 6B–E show the C1s, N1s, O1s, and Fe2p core level XPS spectra. As shown in Fig. 7E, the peaks at the binding energies of 724.8 eV and 711.6 eV are attributed to the Fe 2p^1/2^ and Fe 2p^3/2^ binding energies of Fe_2_O_3_. In addition, the satellite peak at the binding energy of 718.3 eV is provided by Fe^3+^ 2p^3/2^. The results from the XPS spectra indicate that Fe^3+^ and Fe_2_O_3_ were successfully introduced into the MCC-*g*-AM/EDA/PA resin.

### Effect of absorbed dose

Because the yield of free radicals can be significantly affected by the absorbed dose, the effect of the absorbed dose on the grafting yield of AM will be discussed. As shown in Fig. 7, the grafting yield of AM increases substantially with increasing absorbed dose, up to a dose of 30 kGy, and then the grafting yield started to decline at a higher absorbed dose. In view of the above results, the highest grafting rate is 247% when the absorbed dose is 30 kGy. Thus, 30 kGy was selected as the basic research object in the following investigations.

### Effect of pH

**Fig. 8:**
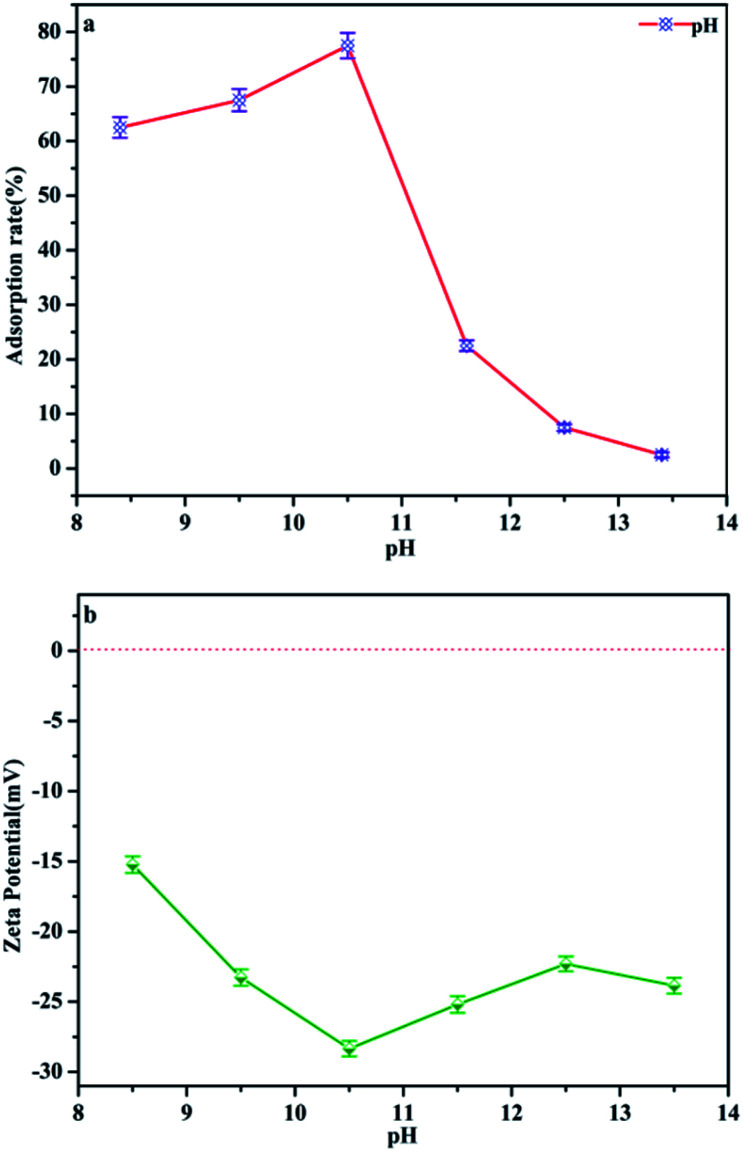
(a) Effect of pH on the phosphate adsorption removal process. (Initial adsorbate concentration: 2 mg L^−1^; adsorption temperature: 25 °C; time = 1 h; adsorbent dosage = 2 g L^−1^). (b) *ζ* potential of MAF as a function of pH.

pH is regarded as one of the most important factors for the adsorption process of water–adsorbent interfaces. Phosphate is polyacidic with three p*K*_a_ values: H_3_PO_4_, H_2_PO_4_^−^ (p*K*_1_ = 2.2), HPO_4_^2−^ (p*K*_2_ = 7.2) and PO_4_^3−^ (p*K*_3_ = 12.4) depending on the solution pH.^25^ In the following, the phosphate adsorption was performed under pH 8.4–13.4 conditions, the dominant anionic forms being HPO_4_^2−^ and PO_4_^3−^, which represents a typical eutrophic ecosystem. Fig. 8a and Table 1 demonstrate the effect of pH on phosphate adsorption onto MAF. The adsorption capacity increases gradually from pH 8.4 to pH 10.4, and reaches a maximum value (77.5%) at pH = 10.4. In the pH range of 8.4–10.4, phosphate anions could be easily adsorbed by Fe^3+^ colloid co-precipitation and chelate to form the inner-sphere surface complex of the MAF adsorbent.^26^ But the adsorption performance dropped faster when the pH increased from 10.4 to 13.4. This may be because the microcrystalline cellulose was partially dissolved in a strong alkaline environment. What's more, as the adsorbent surface is negatively charged as well, the increasing electrostatic repulsion between the negatively charged phosphate ions and the negatively charged MAF would also lead to a decrease in phosphate ion adsorption. In Fig. 8b, the zeta (*ζ*) potential of the adsorbent is electronegative, which means it cannot adsorb phosphate by potential attraction. Thus, it is clear that electrostatic attraction is not a major contributor in this adsorption process and phosphate was removed by chelating with MAF.^27^

**Table 1:** Effect of pH on phosphate adsorption removal rate

pH	Initial concentration phosphate anion/mg L^−1^	Removal rate (%)
8.4	0.75 mg L^−1^	62.5
9.5	0.65 mg L^−1^	67.5
10.5	0.45 mg L^−1^	77.5
11.6	1.55 mg L^−1^	22.5
12.5	1.95 mg L^−1^	7.5
13.4	1.85 mg L^−1^	2.5

### Effect of reaction time and Fe(iii) ion concentration on the chelation process

**Fig. 9:**
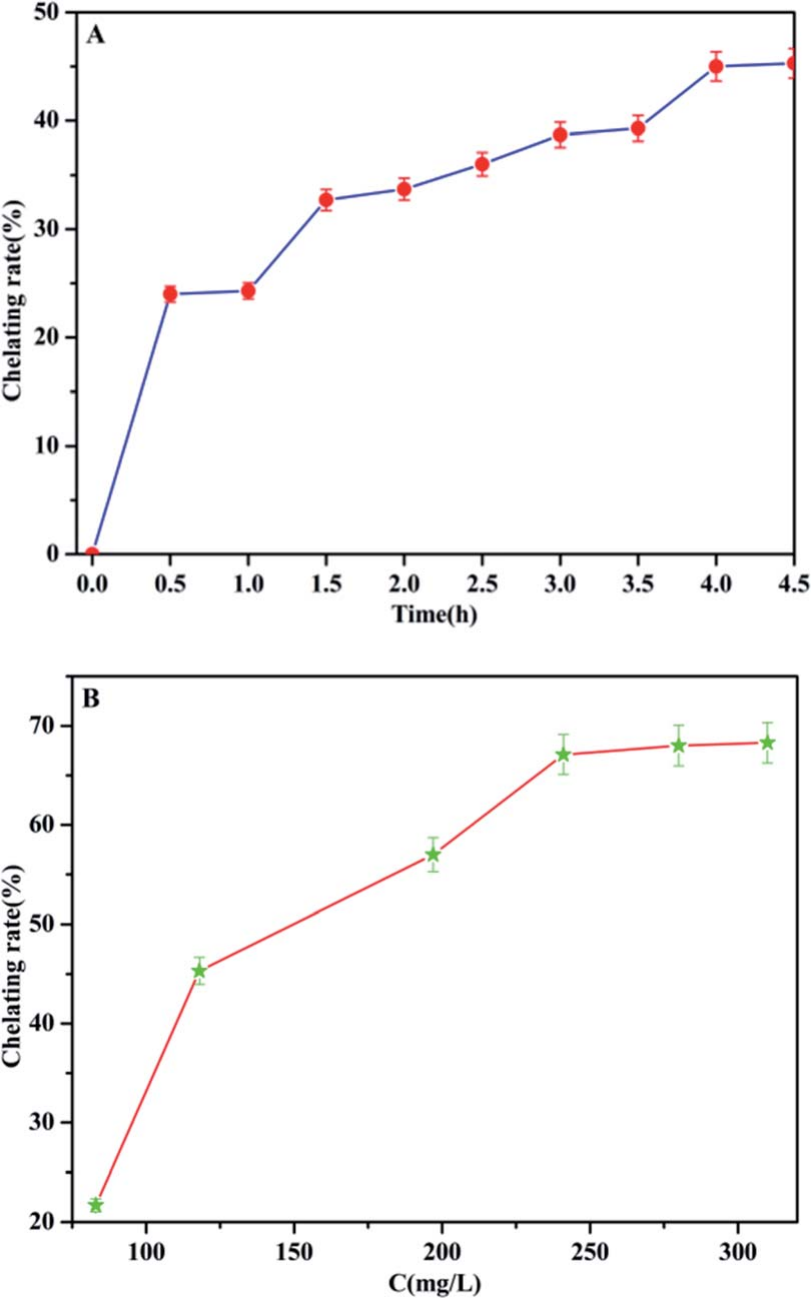
The chelation rate of Fe(iii) by MCC-*g*-AM/EDA/PA as a function of time (A) ([Fe(iii)] = 300 mg L^−1^) and Fe(iii) concentration (B) (contact time = 4 h).

In order to promote the adsorption activity of the phosphate, MCC-*g*-AM/EDA/PA was chelated with Fe(iii) ions, because the Fe(iii) ion is a Lewis acid, which prefers to bind with phosphate (Lewis base). The contact time and chelation of Fe(iii) ions at different initial concentrations was studied at room temperature. As shown in Fig. 9a, the efficiency of Fe(iii) ion chelation increases with the increase of time, and most of the Fe(iii) ions were chelated onto the MCC-*g*-AM/EDA/PA resin surface within 4.0 h, which suggests that the material gets saturated at 4.0 h. Furthermore, the chelation of Fe(iii) ions at different initial concentrations was also investigated and the results are shown in Fig. 9b and Table 2. The Fe(iii) chelation mainly depends on the initial Fe(iii) ion concentration. The resulting chelation rate of Fe(iii) ions at different initial concentrations, over an extensive concentration range of 50–350 mg L^−1^ at room temperature, is shown in Fig. 9b. It was observed that the chelation efficiency of Fe(iii) ions has been increased with the growth of the Fe(iii) ion concentration. But the chelation efficiency tends to be saturated over an Fe(iii) ion concentration of more than 250 mg L^−1^. In order to increase the amount of Fe(iii) ions chelated as much as possible, and reduce the concentration of Fe(iii) ions in the residual liquid, a 300 mg L^−1^ Fe(iii) ion solution was selected as the basic research object in the following investigations.

**Table 2:** Effects of Fe^3+^ concentration on the chelation amount and chelation rate

Fe^3+^ concentration before chelation mg L^−1^	Fe^3+^ concentration after chelation mg L^−1^	Chelation amount mg L^−1^	Chelation rate (%)
83	65	18	21.7
118	65	53	45.3
197	85	112	57.0
241	55	186	67.1
280	90	190	68.1
310	98	212	68.3

### Comparing the adsorption performances between MAF and different intermediate systems

The adsorption behaviors of the MAF adsorbent and its intermediate systems have also been studied to demonstrate the effects of chemical modification. About 0.5 g of adsorbent was added into 100 mL of a 250 mg L^−1^ initial phosphate solution (pH = 10.4) in an iodine flask at room temperature. Then, the reaction mixture was placed onto a thermostat shaker for 1 h, filtered, and the final phosphate concentration was measured. Table 3 records the adsorption rates of the different samples. MCC particles have a lower adsorption rate (14.5%). This may be physical adsorption caused by the presence of micropores on the surface of MCC. The microspheres of MCC grafted with AM show almost no absorption. The amino and carboxyl groups were introduced successively onto the MCC-*g*-AM by reaction modification of EDA and PA to MCC-*g*-AM, and the main reason for this was assumed to be electrostatic interactions,^28^ which makes the adsorption rate increase from 20.7% to 37.3%. Chemical modification contributes to the adsorption of phosphate, especially after the chelation of Fe(iii) ions, where the adsorption of phosphate could reach 89.7%, and this is attributed to the preference of Fe(iii) ions (Lewis acid) bonded with phosphate (Lewis base).^29^

**Table 3:** Adsorption removal rate of phosphates for different adsorbents (time = 1.0 h, *C* = 1.0 mg L^−1^)

Samples	Adsorption removal rate (%)
MCC	14.5%
MCC-*g*-AM	Almost no adsorption
MCC-*g*-AM/EDA	20.9%
MCC-*g*-AM/EDA/PA	37.3%
MAF	89.7%

### Adsorption behaviors of the MAF adsorbent

**Fig. 10:**
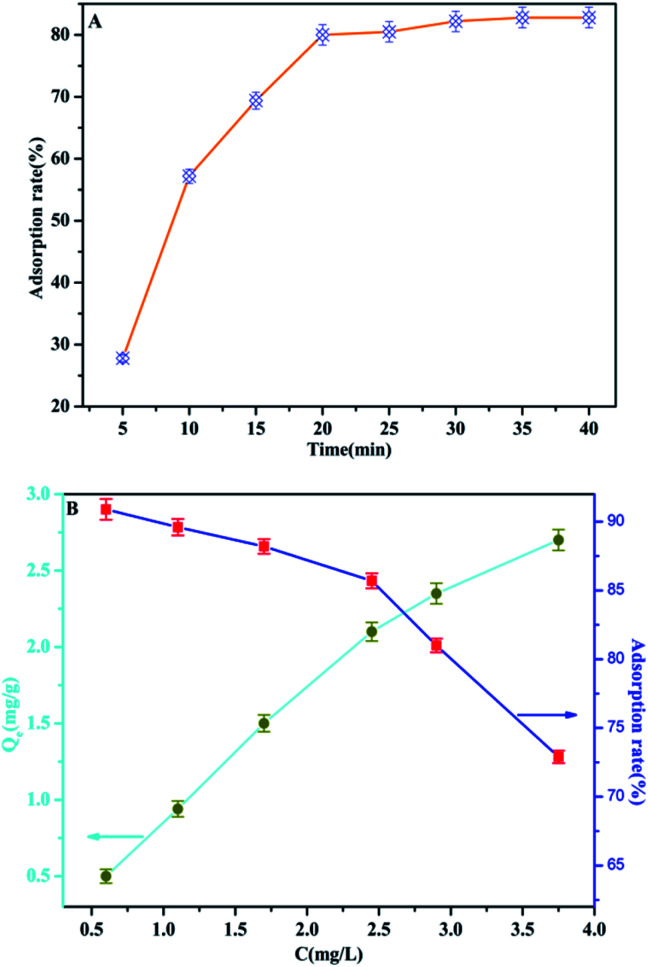
(A) The adsorption rate of phosphate by MAF as a function of time (*C* = 2.0 mg L^−1^); (B) the adsorption capacities and adsorption rate for phosphate on MAF at different phosphate concentrations (time = 40 min).

In order to optimize the minimum contact time for maximum phosphate adsorption onto the interfaces of the MAF adsorbent, the following study was carried out. It can be seen from Fig. 10a that the adsorption rate increases rapidly over 20 min, but the adsorption rate dropped significantly after 20 min, and the adsorption capacity tended to be saturated after 35 min. The adsorption of phosphate at different initial concentrations was studied at room temperature. As shown in Fig. 10b, the adsorption efficiency of the MAF adsorbent is quite considerable, and the adsorption effect is better at low concentration (less than 2.5 mg L^−1^), where it can reach more than 85%. Furthermore, the adsorption of phosphate onto the interfaces of the MAF adsorbent increases with the increase of the phosphate concentration, and the adsorption capacities are increased from 0.5 to 2.7 mg g^−1^ at the initial phosphate concentration range from 0.5 to 3.7 mg g^−1^, but the adsorption efficiency decreases with increasing concentration of phosphate.

### Effect of competing anions on phosphate adsorption

**Fig. 11:**
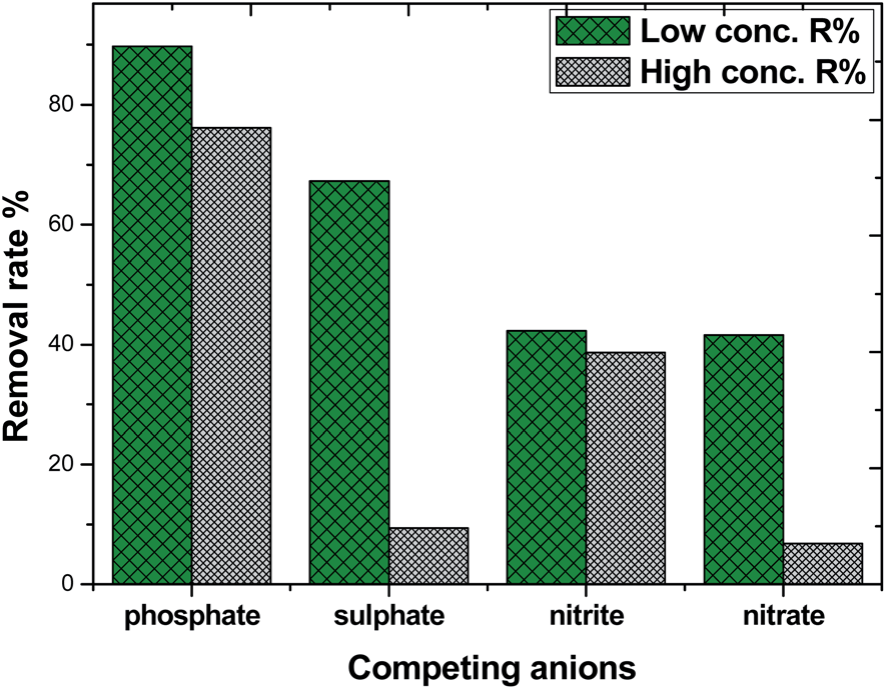
Effect of competing anions on phosphate adsorption.

Considering the complexity and diversity of the components in actual wastewater, in order to further study the selective adsorption capacity of MAF for phosphate, several anionic ions were added to the phosphate solution. The effect of competing ions at two concentration levels at pH 10.4 is shown in Fig. 11. The adsorption removal rate of phosphate on MAF was higher than that of the other competing ions at both concentration levels. The adsorption removal rate order was phosphate > sulphate > nitrite ≈ nitrate, and the adsorption of phosphate was 89.7% at a low concentration level. However, at a high concentration level, the adsorption removal rate order was changed to phosphate > sulphate > nitrite > nitrate, and this time, the adsorption of phosphate was only 76.5%. The competition experiment of sulphate, nitrite, and nitrate on the phosphate removal rate revealed further the formation of inner-sphere surface complexes in the adsorption process of phosphate.^20,30^ These results also show that the MAF adsorbent is suitable for the removal of low concentration levels of phosphate.

### Desorption/regeneration of adsorbent

**Fig. 12:**
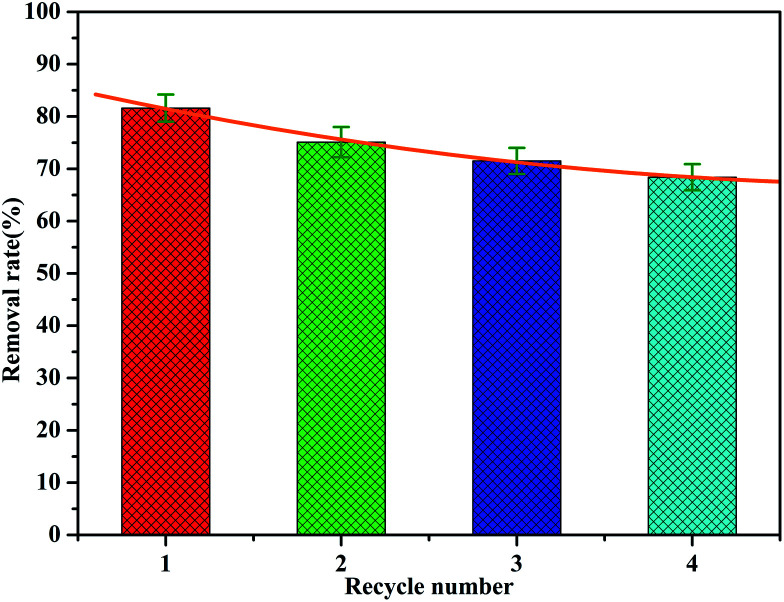
Cyclic adsorption in batch experiments for MAF.

The recovery and regeneration of an adsorbent are important factors for any adsorption process. In the regeneration study, the recovered adsorbent was first washed with distilled water several times, and then added to HCl (0.1 mol L^−1^) and stirred for 1 h. Four experiment cycles were conducted to evaluate the reusability of the adsorbent with the initial concentration of 2.0 mg L^−1^ and adsorbent dosage of 1 g L^−1^. The cyclic adsorption results are illustrated in Fig. 12. The phosphate removal efficiency of MAF was about 81.6% for the first cycle during 1 h. The adsorption rate decreases as the number of cycles increases, but the rate of decrease slows down. Even after four cycles, the desorption efficiency was about 68%. This indicates that a small fraction of active sites were irreversible after regeneration, which is probably because of the strong bonding between MAF and phosphate.

### Possible mechanism for phosphate adsorption onto MAF

**Fig. 13:**
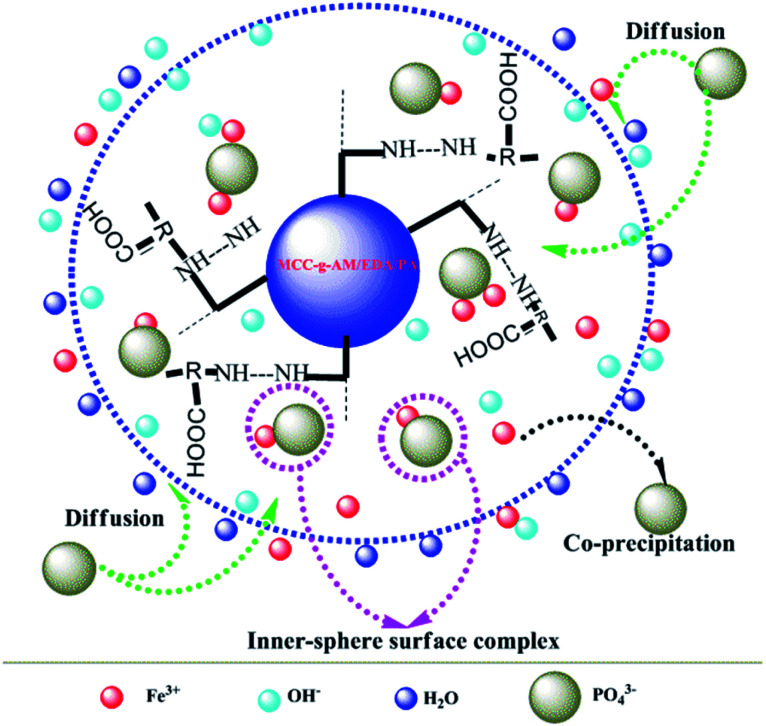
Possible mechanism for phosphate adsorption onto MAF (8.4 ≤ pH ≤ 13.4).

Based on the results of the characterization of MAF and the effect of pH and coexisting anions on phosphate adsorption, the possible phosphate adsorption mechanisms onto the interfaces of MAF can be deduced as shown in Fig. 13. Phosphate removal occurs either *via* precipitation, or inner-sphere surface complexation reaction under alkaline conditions.^26,28^ In the pH test range, Fe^3+^ and PO_4_^3−^ can generate the product FePO_4_ by co-precipitation.^31,32^ Meanwhile, an inner-sphere surface complex (Fe–O–P) between phosphate^33,34^ and Fe_2_O_3_ may be formed in the process of adsorption, which has also been ascertained by TG, XPS, *etc.* Overall, the above-described results demonstrate that the phosphate desorption process by MAF is related to precipitation and formation of an inner-sphere complex in the pH range from 8.4 to 13.4.^35^

## Experimental section

### Preparation of MCC-*g*-AM

MCC-*g*-AM resin was fabricated by a similar procedure to that reported in the literature:^21^ MCC (5.0 g) was pre-irradiated in a vacuum under the cooling of dry-ice by a 1 MeV electron accelerator (Wasik Associates, USA), and 50.0 g solutions (20 w% AM) were injected into three flasks after the sample was bubbled with nitrogen in order to eliminate the presence of oxygen in the ampoule. Subsequently, the pre-irradiated MCC was added into the above emulsion solution at 50 °C for 2 h with continuous nitrogen sparging. After the reaction, the samples were washed excessively with acetone and water to remove unreacted reagents and byproducts, and then freeze-dried for 24 h. The grafting yield (GY) was calculated using eqn (1):^22^1
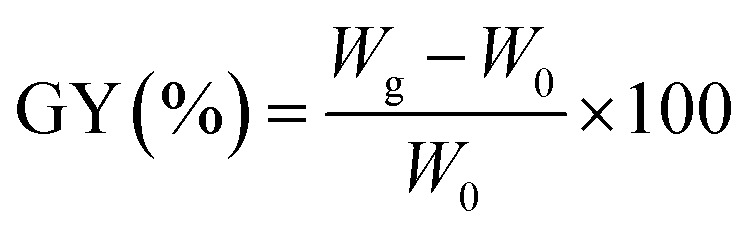
where *W*_0_ (g) and *W*_g_ (g) are the weights of MCC and MCC-*g*-AM, respectively.

### Chemical modification of MCC-*g*-AM

A specific functional group was introduced into the MCC-*g*-AM resin through chemical treatment of the grafted functional groups *via* refluxing ethylenediamine and phthalic anhydride.^17^ The entire process is illustrated in Fig. 1. First, the MCC-*g*-AM (2.0 g) was immersed into 50 mL of EDA aqueous solution at 100 °C for 8 h, and the products were rinsed with water and ethanol, and then dried at 60 °C under vacuum. After that, the MCC-*g*-AM/EDA resins were refluxed with phthalic anhydride (PA) (w/w = 1 : 1) at 100 °C for 6 h to complete the functionalization and obtain the sample of MCC-*g*-AM/EDA/PA.

### Chelation process of Fe(iii) ions

The chelation was measured by dispersing 2.0 g of MCC-*g*-AM/EDA/PA into 200 mL of Fe(iii) solution at different concentrations under magnetic stirring for 4 h. The Fe(iii) concentration was measured by a GDYS-201 multi-parameter water quality analyzer (Little Swan, China). The chelation efficiency of Fe(iii) was analyzed using eqn (2):^20^2
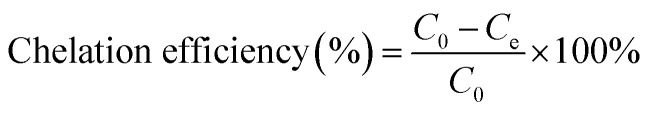
where *C*_0_ (mg L^−1^) and *C*_e_ (mg L^−1^) denote the initial and final concentrations of Fe(iii).

### Bath adsorption experiments of phosphate anions

The adsorption properties of the resins were tested by using sodium phosphate dodecahydrate (Na_3_PO_4_·12H_2_O) with neutral pH at room temperature (25 ± 2 °C). Typically, 0.2 g of MAF resin was dispersed into 100 mL of phosphate solution at different concentrations under oscillation for 40 min to ensure an adsorption–desorption equilibrium. The phosphate concentration was monitored by a GDYS-201M multi-parameter water quality analyzer (Little Swan, China). The adsorption was analyzed using eqn (3):^22^3
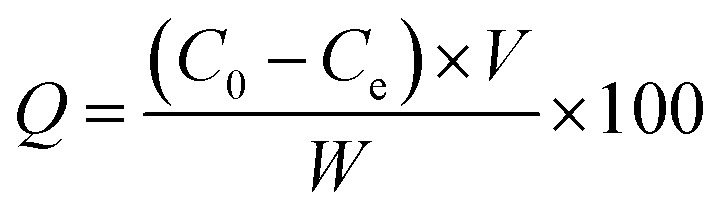
where *C*_0_ (mg L^−1^) and *C*_e_ (mg L^−1^) denote the initial and final concentrations, and *V* (L) and *W* (g) represent the volume of the solution and weight of the adsorbent.

### Characterization

The thermal stability of the powdered samples was investigated on a NETZSCH thermogravimetric analyzer (TG 209F3, Germany). The dried samples were heated from 30 °C to 800 °C at a heating rate of 10°Cmin^−1^ under a nitrogen atmosphere. The crystal structures of the resins were characterized by X-ray powder diffraction (LabX XRD-6100, Shimazdu, Japan). Fourier transform infrared spectra (FTIR) were recorded on a NICOLET 5700 spectrometer (Thermo Fisher Nicolet, America) in the range of 4000–400 cm^−1^. The morphology and microstructure were characterized by scanning electron microscopy (SEM; VEGA-3 SBH, Tescan, Czech Republic). X-ray photoelectron spectroscopic (XPS) measurements were performed using a PerkinElmer PHI 5000C (PE-PHI, US). All of the binding energies were calibrated by using the contaminant carbon (C1s = 284.6 eV) as a reference. The zeta (*ζ*) potential of the adsorbent was tested by particle size & zeta potential analyzers (Nano Brook 90 Plus Pals).

## Conclusion

In summary, MCC-*g*-AM/EDA/PA adsorbent resin has been successfully prepared by an electron beam irradiation method followed by chemical modification. Fe(iii) was successfully introduced into the modified grafted chains of the MCC-*g*-AM/EDA/PA resin and analyzed by FTIR, TG, XRD, SEM and XPS. It was also found that the MAF adsorbent resin shows excellent adsorption performance toward phosphate under alkaline conditions, owing to the synergistic effect between precipitation and the inner-sphere surface complexation reaction. The adsorption efficiency can reach 89.7% at low concentration. The MAF adsorbent has the advantages of being easily separated and recycled in bulk materials, and avoiding secondary pollution. In brief, MAF could be employed as an environmentally friendly adsorbent for phosphate removal in eutrophic water.

## Conflicts of interest

There are no conflicts to declare.

## Supplementary Material

## References

[cit1] El-Naggar M. E., Radwan E. K., El-Wakeel S. T., Kafafy H., Gad-Allah T. A., El-Kalliny A. S., Shaheen T. I. (2018). Int. J. Biol. Macromol..

[cit2] Sun X. B., Zhou Y. L., Zheng X. G. (2020). J. Environ. Chem. Eng..

[cit3] Sowmya A., Meenakshi S. (2013). J. Environ. Chem. Eng..

[cit4] Jia Z. Y., Zeng W., Xu H., Li S. S., Peng Y. Z. (2020). Process Safety and Environmental Protection.

[cit5] Song L., Huo J., Wang X., Yang F., He J., Li C. (2016). Chemical Engineering Journal.

[cit6] Chen X. Y., Liu L. F., Feng Y. W., Wang L. F., Bian Z. F., Li H. X., Wang Z. L. (2017). Mater. Today..

[cit7] Xu M. J., Chen Y., Qin J. T., Feng Y. W., Li W., Chen W., Zhu J., Li H. X., Bian Z. F. (2018). Environ. Sci. Technol..

[cit8] Zhao B. C., Jiang H. B., Lin Z. K., Xu S. F., Xie J., Zhang A. (2019). Carbohydr. Polym..

[cit9] Peng C., Hong Z., Chong W., Ma H., Hu J., Pu Y., Peng L. (2012). J. Hazard. Mater..

[cit10] Dong Z., Yuan W. J., Li Y., Hua R., Zhao L. (2019). Radiat. Phys. Chem..

[cit11] Hao J., Xu S. Y., Xu N. Y., Li D. X., Linhardt R. J., Zhang Z. Q. (2017). Carbohydr. Polym..

[cit12] Fierro A. T., Bauzán M. V., Origoni M. X., Trezza V. M. (2018). SF Journal of Pharmaceutical and Analytical Chemistry.

[cit13] Zango Z. U., Imam S. S. (2018). Nanosci. Nanotechnol..

[cit14] Chen Y. M., Zhu H. X., Xiong J. H., Zhang C. Z., Li Y. H. (2017). J. Bioresour. Bioprod..

[cit15] Liu Y. J., Liu S., Li Z. W., Ma M. G., Wang B. (2018). RSC Adv..

[cit16] Zhuang S. T., Yin Y. A., Wang J. L. (2018). Nucl. Eng. Technol..

[cit17] Senna M. M. H., Abdel-Moneam Y. K., Gamal O. A., Alarifi A. (2013). J. Ind. Eng. Chem..

[cit18] Akkas-Kavaklı P., Kavaklı C., Güven O. (2010). Radiat. Phys. Chem..

[cit19] Barsbay M., Akkas-Kavaklı P., Güven O. (2010). Radiat. Phys. Chem..

[cit20] Akkaş-Kavaklı P., Kavaklı C., Güven O. (2014). Radiat. Phys. Chem..

[cit21] Seko N., Hoshina H., Kasai N., Shibata T., Saiki S., Ueki Y. (2018). Radiat. Phys. Chem..

[cit22] Li Y. S., Qin J. T., Han Y., Du J. F., Dong Z. B., Sun S. F., Liu Y. (2017). Appl. Catal., B.

[cit23] Du X. L., Han Q., Li J. Q., Li H. Y. (2017). J. Taiwan Inst. Chem. Eng..

[cit24] Xing B. B., Chen T. H., Liu H. B., Qing C. S., Xie J. J., Xie Q. Q. (2017). J. Taiwan Inst. Chem. Eng..

[cit25] Borgnino L., Giacomelli C. E., Avena M. J., De Pauli C. P. (2010). Colloids Surf., A.

[cit26] Rout P. R., Bhunia P., Dash R. R. (2015). J. Taiwan Inst. Chem. Eng..

[cit27] Luo X., Liu C., Yuan J., Zhu X., Liu S. (2017). ACS Sustainable Chem. Eng..

[cit28] Yang Y. J., Wang J. J., Qian X. Q., Shan Y. H., Zhang H. P. (2018). Appl. Surf. Sci..

[cit29] Wang J., Wu L. Y., Li J., Tang D. D., Zhang G. K. (2018). J. Alloys Compd..

[cit30] Andrés E., Araya F., Vera I., Pozo G., Vidal G. (2018). Ecological Engineering.

[cit31] Yang Q., Wang X. L., Luo W., Sun J., Zeng G. M. (2018). Bioresour. Technol..

[cit32] Ray J., Jana S., Tripathy T. (2018). Int. J. Biol. Macromol..

[cit33] Mitrogiannis D., Psychoyou M., Baziotis I., Inglezakis V. J., Koukouzas N., Tsoukalas N., Palles D., Kamitsos E., Oikonomou G., Markou G. (2017). Chem. Eng. J..

[cit34] Zhang L., Liu J. X., Guo X. S. (2018). J. Environ. Sci..

[cit35] Li Y., Fu F., Cai W., Tang B. (2019). Powder Technol..

